# Garland Rolling Circle Amplification Mediated Self-Priming Extension Strategy for Sensitive and Label-Free *Pseudomonas aeruginosa* Analysis in Perioperative Period

**DOI:** 10.4014/jmb.2603.03031

**Published:** 2026-05-11

**Authors:** Yuechen Xu, Lingyun Wei

**Affiliations:** 1 Department of Operation Room, The First People’s Hospital of Linping District, Hangzhou City, Zhejiang Province 311100, P. R. China; 2 Department of Anesthesiology, The First People’s Hospital of Chun’an County, Hangzhou City, Zhejiang Province 311700, P. R. China

**Keywords:** *Pseudomonas aeruginosa*, Garland rolling circle amplification, Self-priming, Aptamer, SYBR Green-I

## Abstract

Early and accurate detection of *Pseudomonas aeruginosa* (*P. aeruginosa*) is critically important in perioperative care to prevent severe healthcare-associated infections and guide timely antimicrobial intervention. Herein, we report a novel biosensing strategy for sensitive and label-free detection of *P. aeruginosa* by integrating F23 aptamer-mediated target recognition, garland rolling circle amplification (RCA)-triggered self-priming extension, and SYBR Green I (SG-I)-based fluorescence readout. The capture probe, comprising the F23 aptamer and a primer strand immobilized on magnetic nanoparticles, specifically recognizes *P. aeruginosa* and releases the primer to initiate dumbbell probe circularization and subsequent RCA. The resulting RCA products are cleaved by a nicking endonuclease to generate fragmented DNA, which then hybridizes with a hairpin probe to prime cyclic self-extension reactions, producing abundant double-stranded DNA for SG-I intercalation and fluorescence enhancement. Under optimized conditions, the proposed method achieves a detection limit as low as 2.3 CFU/mL with a wide linear range from 10 to 10^6^ CFU/mL. The assay exhibits excellent specificity against non-target bacteria, robust stability during storage, and satisfactory anti-interference capability in complex clinical matrices. Validation using clinical samples demonstrates excellent agreement with the gold-standard colony counting method while reducing the assay time to less than 2.5 h without requiring nucleic acid extraction or thermal cycling. With its label-free design, isothermal amplification, and operational simplicity, this strategy holds great promise for point-of-care testing in perioperative settings and can be readily adapted for detecting other pathogens by substituting the corresponding aptamer.

## Introduction

Perioperative care plays a pivotal role in ensuring patient safety and recovery, yet it also presents a critical window of vulnerability to healthcare-associated infections [1]. Among the diverse array of pathogens encountered in clinical settings, *Pseudomonas aeruginosa* (*P. aeruginosa*) stands out as a leading cause of postoperative complications, particularly in immunocompromised or critically ill patients [2, 3]. This opportunistic pathogen is frequently implicated in surgical site infections, ventilator-associated pneumonia, and catheter-related bloodstream infections, posing substantial challenges to nursing care and clinical management during the perioperative period [4, 5]. Given its intrinsic and acquired resistance to multiple antibiotics, early and accurate detection of *P. aeruginosa* is essential for guiding timely antimicrobial therapy, implementing effective infection control measures, and improving patient outcomes. Therefore, the development of sensitive and reliable diagnostic strategies tailored to the perioperative setting is of great clinical importance.

Conventional methods for the detection of *P. aeruginosa* primarily rely on culture-based colony counting, which remains the gold standard in clinical microbiology due to its reliability and low cost [6, 7]. However, this technique is time-consuming, typically requiring 24–48 hours for visible colony formation, and is limited by its inability to detect viable but non-culturable (VBNC) states of bacteria. Moreover, culture-based assays often suffer from low sensitivity in complex clinical specimens, where bacterial loads may be minimal yet clinically significant. As an alternative, polymerase chain reaction (PCR)-based techniques offer enhanced sensitivity and specificity by amplifying bacterial DNA [8, 9]. Nevertheless, PCR requires intricate nucleic acid extraction, thermal cycling equipment, and trained personnel, which restricts its application in point-of-care or resource-limited settings, particularly during the perioperative period when rapid diagnosis is critical.

In recent years, aptamer-based biosensing platforms have attracted considerable attention due to their high specificity, ease of synthesis, and chemical stability [10]. Aptamers are short, single-stranded oligonucleotides that can fold into unique three-dimensional structures, enabling them to bind target molecules with affinity comparable to that of antibodies [11]. Specifically, the F23 aptamer has been identified as a high-affinity ligand for *P. aeruginosa*, offering a promising recognition element for pathogen detection [12-14]. For instance, Xie *et al*. developed a fluorescence assay based on aptamer-functionalized magnetic nanoparticles for selective capture and detection of *P. aeruginosa*, demonstrating the potential of aptamer-based strategies in bacterial analysis [15]. However, such approaches often require integration with isothermal amplification techniques to achieve sufficient sensitivity for clinical applications. Among these, rolling circle amplification (RCA) has gained considerable interest due to its simple operation, high amplification efficiency, and capability to generate long single-stranded DNA under isothermal conditions [16-19]. Despite its advantages, RCA-based bacterial detection still faces challenges in achieving adequate sensitivity, particularly in the absence of secondary amplification steps [20]. Furthermore, many existing RCA assays rely on fluorescent dye labeling, which is susceptible to environmental factors such as pH and temperature fluctuations, compromising signal stability and reproducibility [21]. Therefore, there is an urgent need to develop a highly sensitive, label-free detection strategy for *P. aeruginosa* that integrates the specificity of aptamer recognition with the amplification power of RCA, while circumventing the limitations associated with conventional labeling techniques.

To address the aforementioned limitations, we herein propose a novel biosensing strategy that integrates specific recognition of *P. aeruginosa* by the F23 aptamer, signal amplification via garland rolling circle amplification (which is fundamentally a dumbbell probe RCA but emphasizes the garland-like tandem-repeat architecture of the resulting products, whereas the term “dumbbell probe RCA” typically highlights the circularization process of the template; both refer to the same RCA mechanism) -mediated self-priming extension, and label-free fluorescence detection using SYBR Green I (SG-I). This approach enables direct and sensitive analysis of *P. aeruginosa* without the need for cumbersome sample pretreatment or fluorescent dye labeling. The garland RCA strategy, which generates tandem-repeat DNA products with self-priming capability, facilitates efficient signal amplification under isothermal conditions, thereby endowing the method with a high sensitivity. Meanwhile, the use of SG-I as a signal reporter allows for real-time monitoring of amplification products in a label-free manner, circumventing the stability issues associated with covalently labeled fluorophores. As a result, the proposed method achieves a detection limit as low as 2.3 CFU/mL for *P. aeruginosa*, with a linear response spanning five orders of magnitude, demonstrating superior analytical performance compared to existing aptamer-based assays. Moreover, the assay is straightforward, rapid, and amenable to point-of-care applications, making it particularly suitable for perioperative infection monitoring. Given its high sensitivity, simplicity, and adaptability, this strategy holds great promise for clinical diagnosis, food safety inspection, and environmental surveillance of *P. aeruginosa*, and can be readily extended to detect other pathogens by substituting the corresponding aptamers.

## Experimental Section

### Reagents and Materials

All oligonucleotides used in this study were synthesized and purified by Sangon Biotech Co., Ltd. (China). The sequences were as listed in Table S1. T4 DNA ligase, phi29 DNA polymerase, nicking endonuclease Nt.BbvCI, and corresponding reaction buffers were purchased from New England Biolabs (China). SYBR Green I (SG-I) nucleic acid gel stain was obtained from Thermo Fisher Scientific (China). Magnetic nanoparticles (MNPs) were purchased from Allrumano Technology Co., Ltd. (China). 1-Ethyl-3-(3-dimethylaminopropyl) carbodiimide (EDC) and N-hydroxysuccinimide (NHS) were obtained from Aladdin Biochemical Technology Co., Ltd. (China). Bovine serum albumin (BSA) was purchased from Solarbio Science & Technology Co., Ltd. (China). Luria-Bertani (LB) broth and agar were obtained from Hopebio Biotechnology Co., Ltd. (China). All other chemicals were of analytical grade and used without further purification. Deionized water (18.2 MΩ·cm) used throughout the experiments was produced by a ULUPURE water purification system (China). This study was approved by the Medical Ethics Committee of The First People’s Hospital of Chun’an County (Approval No. 2024-04-03-12).

### Assembly and Immobilization of Capture Probe on MNPs

The carboxyl-modified MNPs (5 mg) were washed three times with MES buffer (25 mM, pH 5.5) and re-suspended in 500 μL of the same buffer. Subsequently, 250 μL of EDC (50 mM) and 250 μL of NHS (50 mM) were added to activate the carboxyl groups, and the mixture was incubated at 37°C for 30 min with gentle shaking. After magnetic separation, the activated MNPs were washed with Tris-HCl buffer (20 mM, pH 7.4) and re-suspended in 500 μL of the same buffer. Then, 2 nmol of amino-modified strand “1” was added, and the mixture was incubated at 37°C for 2 h with continuous shaking to allow covalent conjugation. The resulting “1”@MNPs were collected by magnetic separation, washed three times with Tris-HCl buffer to remove unbound strands, and blocked with BSA (1 mg/mL) at 37°C for 30 min to minimize nonspecific adsorption.

For capture probe assembly, the “1”@MNPs were re-suspended in hybridization buffer (Tris-HCl 20 mM, NaCl 300 mM, MgCl_2_ 5 mM, pH 7.4) containing 2 nmol of F23 aptamer. The mixture was incubated at 37°C for 1 h to facilitate hybridization between the aptamer and strand “1”. The resulting capture probe@MNPs were washed three times with Tris-HCl buffer to remove excess aptamer and stored at 4°C until further use.

### Detection Procedure

For *P. aeruginosa* detection, 50 μL of capture probe@MNPs suspension (5 mg/mL) was mixed with 200 μL of bacterial sample at various concentrations and incubated at 37°C for 45 min with gentle shaking to allow target recognition and strand displacement. After magnetic separation, the supernatant containing the released “1” strand was collected. To this supernatant, 50 μL of dumbbell probe (DP, 100 nM), 10 U of T4 DNA ligase, and 1× ligation buffer were added, and the mixture was incubated at 37°C for 30 min to circularize the DP. Subsequently, 5 U of phi29 DNA polymerase, 500 μM dNTPs, and 1× polymerase buffer were introduced, and rolling circle amplification was carried out at 30°C for 60 min. The RCA products were then treated with 10 U of nicking endonuclease Nt.BbvCI at 37°C for 30 min to generate fragmented DNA (Rp), followed by thermal inactivation at 80°C for 10 min. Next, 50 μL of hairpin probe (Hp, 500 nM) and 5 U of phi29 DNA polymerase were added to initiate the self-priming extension reaction at 30°C for 40 min. Finally, 10 μL of SYBR Green I (10× diluted) was added, and the mixture was incubated at room temperature for 10 min in the dark. Fluorescence spectra were recorded using an F-380 fluorescence spectrophotometer (Tianjin Gangdong Sci. & Tech. Development Co. Ltd., China) with excitation at 494 nm and emission collected from 510 to 600 nm. The fluorescence intensity at 525 nm was used for quantitative analysis. All experiments were performed in triplicate.

## Results

### Working Mechanism of the Proposed Biosensing Strategy

**Fig. 1:**
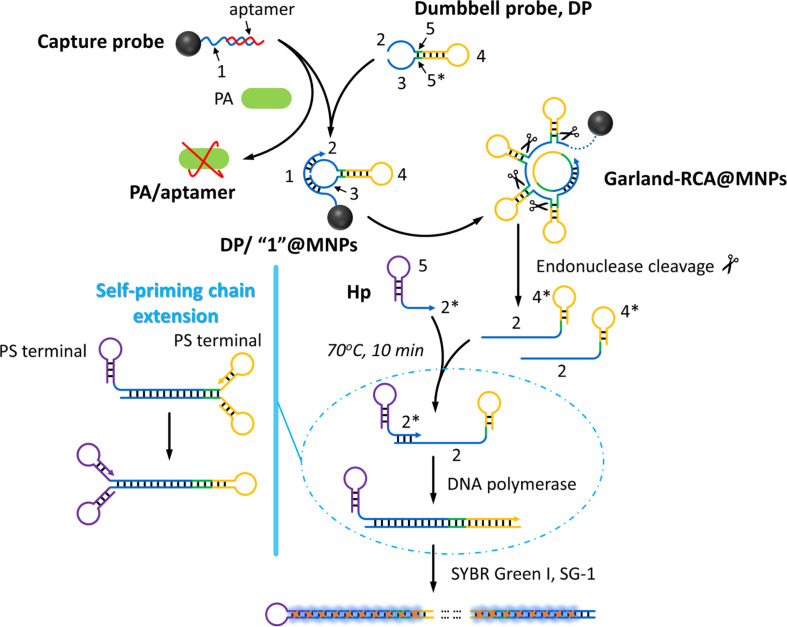
Working mechanism of the proposed method for sensitive and label-free *P. aeruginosa* analysis.

The working principle of the proposed biosensing strategy for *P. aeruginosa* detection is schematically illustrated in Fig. 1. The capture probe, consisting of a hybridized assembly of two oligonucleotide strands (designated as strand “1” and the F23 aptamer), is immobilized onto the surface of magnetic nanoparticles (MNPs) to enable specific recognition of target bacteria and subsequent signal amplification. In the presence of *P. aeruginosa*, the aptamer component preferentially binds to the bacterial surface, undergoing a conformational rearrangement that liberates the “1” portion of the capture probe. The exposed “1”@MNPs then serve as a primer to recognize and hybridize with the stem region (comprising “1” and “3”) of a dumbbell probe (DP), facilitating its circularization upon ligation by T4 DNA ligase. Notably, the DP is designed to contain a recognition site for a nicking endonuclease; however, in the absence of the enzyme, the circularized DP remains intact and un-cleaved.

Subsequently, using the tethered “1” as a primer, rolling circle amplification is initiated in the presence of DNA polymerase, generating elongated DNA products consisting of tandem-repeat hairpin structures containing the “4*” sequence. Following magnetic separation to remove unbound components, a nicking endonuclease is introduced to specifically recognize and cleave the “5*” region embedded within the RCA products. This cleavage event releases fragmented DNA products (Rp) that harbor both the “4*” and “2” sequences. After thermal inactivation of the endonuclease, a hairpin probe (Hp), which contains complementary “5*” and “2*” domains, is added to the reaction system. It is important to note that the “4*” and “5*” sequences are designed with self-complementary ends, predisposing them to intramolecular hybridization.

The “2*” region of Hp hybridizes with the “2” sequence present on the Rp fragments, thereby priming a new round of DNA polymerization using Rp as the template. This extension reaction generates the “4” sequence, which exhibits a strong tendency to self-hybridize due to its intrinsic complementarity. The resulting self-primed structure subsequently initiates another round of amplification, driving a cyclic cascade of extension events. Through this self-perpetuating amplification mechanism, long tandem double-stranded DNA products are progressively accumulated. Finally, SYBR Green I (SG-I) is introduced as a signal reporter, which intercalates specifically into the double-stranded regions of the amplification products, generating a significantly enhanced fluorescence output that correlates quantitatively with the concentration of target *P. aeruginosa*. This elegantly designed mechanism integrates target recognition, signal amplification, and label-free fluorescence detection into a unified platform, offering high sensitivity and operational simplicity.

### Validation of the Assay Construction and Feasibility

**Fig. 2:**
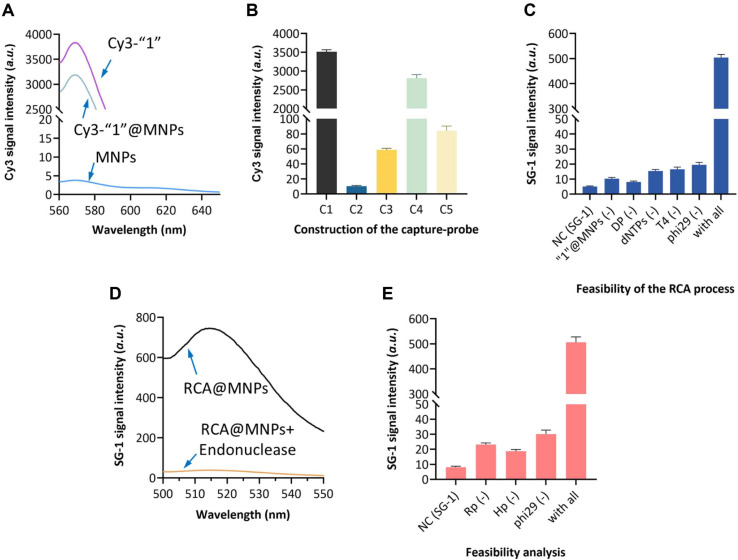
Assembly of the capture probe and feasibility analysis of the method. (**A**) Fluorescence spectrum of the Cy3 labeled “1” sequence before and after being labeled on the surface of MNPs. (**B**) Cy3 signal intensity of the Cy3 labeled “1” sequence during the construction of the capture probe. (**C**) SG-1 signal intensity of the RCA product when essential components existed or not. (**D**) SG-I signal intensity of the RCA product when endonuclease was added or not. (**E**) SG-I signal intensity of the method when essential components existed or not.

To verify the successful immobilization of the “1” strand onto the magnetic nanoparticles (MNPs), fluorescence measurements were conducted using Cy3-labeled “1” (Cy3-“1”). As shown in Fig. 2A, after incubation of Cy3-“1” with carboxyl-modified MNPs and subsequent magnetic separation, the re-suspended MNPs exhibited a strong fluorescence signal, indicating efficient conjugation of the “1” strand onto the MNP surface. In contrast, the control group without EDC/NHS activation showed negligible fluorescence, confirming that the immobilization was achieved via covalent amide bonding rather than nonspecific adsorption.

The assembly of the capture probe and its responsiveness to target bacteria were subsequently investigated using a fluorescence resonance energy transfer (FRET)-based strategy. The aptamer strand was labeled with Cy3 at its 5’ end (C1), while the “1” strand was modified with a Black Hole Quencher (BHQ) at its 3’ end (C2). As illustrated in Fig. 2B, upon hybridization of the Cy3-aptamer with BHQ-“1” on the MNP surface, a significant decrease in fluorescence intensity was observed due to the close proximity between the fluorophore and quencher (C3), confirming successful formation of the capture probe complex. However, when target *P. aeruginosa* was introduced into the system, a marked recovery of fluorescence was detected (C4). This result demonstrates that the aptamer preferentially binds to the bacteria, leading to the dissociation of the Cy3-aptamer from the BHQ-“1”@MNPs and subsequent restoration of the fluorescence signal, thereby validating the target recognition capability of the designed capture probe. When the capture probe is mixed with interfering bacteria, the recorded fluorescence signal intensity remained in low level (C5).

The occurrence of rolling circle amplification was then evaluated using SYBR Green I (SG-I) as a fluorescence reporter. SG-I is a DNA intercalating dye that exhibits strong fluorescence enhancement upon binding to double-stranded DNA, making it particularly suitable for monitoring amplification products containing duplex structures. As shown in Fig. 2C, when the “1”@MNPs, dumbbell probe, T4 DNA ligase, phi29 DNA polymerase, and dNTPs were all present in the reaction system, a dramatic increase in SG-I fluorescence intensity was observed. In contrast, control groups lacking any of these essential components showed only background fluorescence levels. This result confirms that the circularization of the dumbbell probe and subsequent RCA proceed as designed, generating abundant hairpin-structured DNA products that provide binding sites for SG-I intercalation.

To validate the cleavage efficiency of the nicking endonuclease, the RCA products immobilized on MNPs were incubated with the enzyme, followed by magnetic separation. The fluorescence intensity of the supernatant was measured using SG-I before and after enzyme treatment. As depicted in Fig. 2D, a substantial decrease in fluorescence signal was observed after nicking endonuclease digestion compared to the untreated control. This reduction indicates successful cleavage of the “5*” region within the RCA products, releasing fragmented DNA (Rp) into the supernatant and leaving the MNP-bound remnants with diminished SG-I binding capacity. The result confirms the specific and efficient cleavage activity of the nicking endonuclease, which is essential for initiating the subsequent self-priming amplification cascade.

Finally, the signal output mediated by self-priming extension was examined. As shown in Fig. 2E, when the Rp fragments, hairpin probe (Hp), and DNA polymerase were incubated together, a significant fluorescence enhancement was observed upon addition of SG-I. By contrast, reactions omitting any of these components failed to produce a notable signal increase. This finding demonstrates that the Hp hybridizes with the Rp fragments via the “2”/ “2*” interaction, priming DNA polymerization that generates the “4” sequence. The self-hybridization propensity of “4” then triggers cyclic extension events, ultimately producing long double-stranded DNA products that are effectively recognized by SG-I. Collectively, these results validate the successful construction of each functional module and confirm the overall feasibility of the proposed biosensing strategy for *P. aeruginosa* detection.

### Analytical Performance of the Proposed Biosensing Strategy

To achieve optimal detection performance, key experimental parameters were systematically investigated, including the concentration of phi29 DNA polymerase, dumbbell probe (DP), and hairpin probe (Hp), as well as the total reaction time. As shown in Fig. S1-S4 (see Electronic Supporting Material), the optimal conditions were determined as follows: 2 U/μL phi29 DNA polymerase, 100 nM DP, 500 nM Hp, and a total reaction time of 150 min. These optimized parameters were employed in all subsequent experiments.

**Fig. 3:**
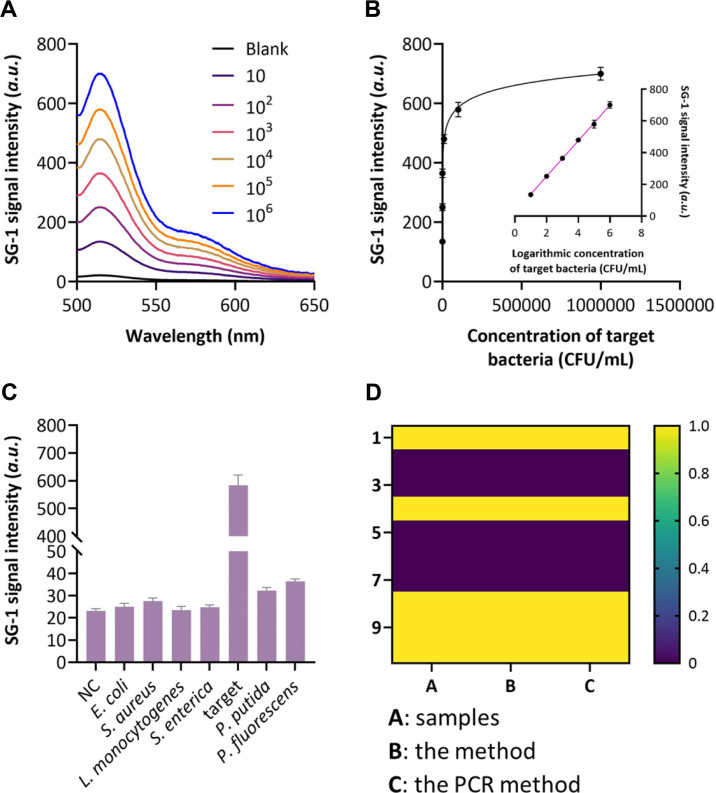
Analytical performance of the method. (**A**) Fluorescence spectrum of the method when detecting different concentrations of target bacteria. (**B**) Correlation between the calculated SG-I signal intensity and the concentration of target bacteria. (**C**) SG-I signal intensity of the method when detecting target bacteria and interfering bacteria. (**D**) Distinguish capability of the method and the PCR method to target bacterial from 10 samples.

Under the optimal conditions, the sensitivity of the proposed method for *P. aeruginosa* detection was evaluated using a series of bacterial concentrations. As depicted in Fig. 3A, the fluorescence intensity of SYBR Green I increased progressively with rising concentrations of *P. aeruginosa* ranging from 10 CFU/mL to 10^6^ CFU/mL. A linear correlation was observed between the fluorescence intensity and the logarithm of bacterial concentration (Fig. 3B), with a regression equation of F = 3.12 lg C + 54.3 (R^2^ = 0.995), where F represents the fluorescence intensity and C is the concentration of *P. aeruginosa* (CFU/mL). The limit of detection (LOD) was calculated to be 2.3 CFU/mL based on three times the standard deviation of the blank measurements (n = 10), demonstrating the high sensitivity of the proposed strategy.

The specificity of the method was further assessed by challenging the system with various non-target bacterial strains, including *Escherichia coli* (*E. coli*), *Staphylococcus aureus* (*S. aureus*), *Listeria monocytogenes* (*L. monocytogenes*), *Salmonella enterica* (*S. enterica*), *Pseudomonas putida* (*P. putida*), and *Pseudomonas fluorescens* (*P. fluorescens*) all at a concentration of 10^5^ CFU/mL. As shown in Fig. 3C, a significantly higher fluorescence signal was observed for *P. aeruginosa* compared to the non-target bacteria, which exhibited fluorescence intensities comparable to the blank control. These results confirm the excellent selectivity of the F23 aptamer-based recognition mechanism, ensuring that the signal amplification cascade is triggered only in the presence of the target pathogen.

To further validate the reliability of the proposed method, its detection results were compared with those obtained by conventional PCR analysis. As illustrated in Fig. 3D, both methods demonstrated excellent consistency in distinguishing *P. aeruginosa* from other bacterial species, with positive signals exclusively observed for the target pathogen. Notably, while PCR required laborious nucleic acid extraction, thermal cycling equipment, and approximately 3-4 h to complete the analysis, the proposed method achieved equivalent specificity without the need for DNA extraction or thermal cycling, with a total assay time of less than 2.5 h. This operational simplicity, combined with the elimination of thermocycling requirements, renders the proposed strategy particularly advantageous for point-of-care applications in perioperative settings where rapid and user-friendly diagnostic tools are urgently needed.

### Clinical Applicability and Method Validation

**Fig. 4:**
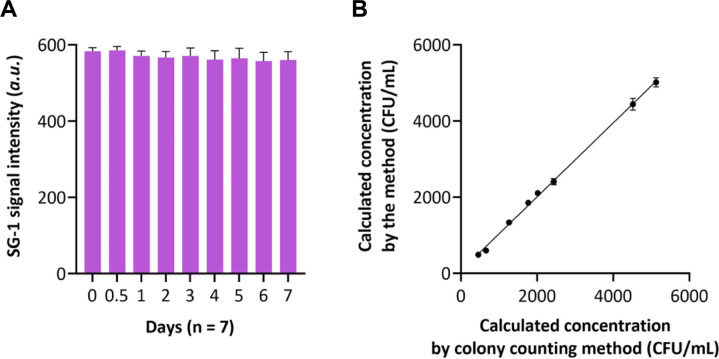
Clinical application potential of the method for target bacteria analysis. (**A**) SG-I signal intensity of the method for the detection of 10^5^ CFU/mL of target bacteria when the components were incubated with different duration. (**B**) Correlation between the calculated target concentration by the method and the traditional colony counting method.

To evaluate the storage stability of the proposed biosensing strategy, the prepared capture-probe@MNPs and associated reagents were stored at -20°C for different durations ranging from 1 to 7 days. At each time point, the system was employed to detect a fixed concentration of *P. aeruginosa* (1000 CFU/mL). As shown in Fig. 4A, the fluorescence signals obtained across the seven days exhibited minimal variation, with relative standard deviations (RSDs) below 5%, indicating that the key biological components retain their functional integrity during frozen storage. This excellent stability underscores the potential of the method for practical applications where reagent storage and transport are necessary considerations.

The anti-interference capability of the proposed method was assessed through recovery experiments in complex sample matrices. Given the intended perioperative application, human serum samples were selected as representative clinical specimens to evaluate matrix effects. Serum samples were spiked with three different concentrations of *P. aeruginosa* (50, 500, and 5000 CFU/mL) and analyzed using the developed method. As summarized in Table 1, the recovery rates ranged from 97.4% to 103.6%, with RSD values below 5.5% for all tested concentrations. These satisfactory recoveries demonstrate that the method possesses robust anti-interference capacity, effectively resisting the influence of complex biological matrices and maintaining accurate quantification of target bacteria.

To further validate the clinical applicability of the proposed strategy, a comparative study was conducted using ten clinical samples (including wound swabs, sputum, and blood samples) collected from postoperative patients. Each sample was simultaneously analyzed by the proposed method and the conventional colony counting method, which serves as the gold standard for bacterial detection. The results are presented in Fig. 4B, showing excellent agreement between the two methods across all tested samples. Statistical analysis revealed no significant difference between the two methods (paired t-test, *p* > 0.05), confirming the reliability and accuracy of the proposed biosensing strategy for clinical sample analysis. Notably, while colony counting required 24-48 h to obtain results, the proposed method completed the entire analysis within 2.5 h, offering a substantial advantage in time efficiency without compromising accuracy. This rapid turnaround time is particularly valuable in perioperative settings where timely clinical decisions are critical for patient management and infection control.

## Discussion

In this work, we have developed a label-free biosensing strategy for *P. aeruginosa* detection by integrating F23 aptamer-mediated recognition, garland rolling circle amplification (RCA)-triggered self-priming extension, and SYBR Green I (SG-I) fluorescence readout. The results presented above demonstrate that the proposed method achieves high sensitivity (LOD = 2.3 CFU/mL), excellent specificity, and satisfactory performance in complex clinical matrices. Several aspects of this strategy merit further discussion in the context of existing detection approaches and practical perioperative applications.

Compared with conventional culture-based methods, which typically require 24–48 h for reliable colony counting, our assay yields results within 2.5 h without the need for nucleic acid extraction or thermal cycling. This rapid turnaround time is particularly advantageous in perioperative settings, where timely antimicrobial intervention can significantly reduce the risk of surgical site infections and other healthcare-associated complications. Furthermore, while PCR-based techniques offer high sensitivity, they depend on expensive thermal cyclers and trained personnel, limiting their point-of-care utility. In contrast, the isothermal nature of our RCA-based amplification cascade, combined with magnetic separation, simplifies the workflow and reduces equipment requirements. Furthermore, our method avoids the need for expensive thermal cyclers and nucleic acid extraction kits, substantially lowering the per-test cost relative to PCR-based assays, and offers a more favorable cost–time balance than culture-based methods due to its dramatically shorter turnaround time. The use of SG-I as a label-free fluorescent reporter circumvents the stability issues associated with covalently labeled fluorophores, which are often sensitive to pH, temperature, and photobleaching, thereby enhancing assay robustness and reproducibility.

The garland RCA-mediated self-priming extension strategy represents a key innovation that distinguishes our method from conventional RCA-based biosensors. Traditional RCA generates long single-stranded DNA products that require secondary amplification or labeled probes to achieve sufficient sensitivity for low-abundance targets. In our design, the nicking endonuclease-cleaved RCA products (Rp) hybridize with a hairpin probe (Hp) and initiate a cyclic self-priming extension reaction, producing abundant double-stranded DNA that is readily recognized by SG-I. This cascade amplification mechanism not only enhances the overall signal gain but also maintains isothermal conditions throughout the entire process. Notably, the self-priming property eliminates the need for exogenous primers in the later amplification stages, reducing assay complexity and potential nonspecific background.

Specificity is another critical parameter for pathogen detection in clinical samples. The F23 aptamer has been previously reported to bind *P. aeruginosa* with high affinity and selectivity. Our results confirm that the aptamer-based capture probe effectively distinguishes the target from common interfering bacteria, including *E. coli*, *S. aureus*, *L. monocytogenes*, and *S. enterica*. This high specificity is essential for avoiding false-positive results in polymicrobial clinical specimens, such as wound swabs and sputum from postoperative patients. Moreover, the recovery experiments in human serum samples (97.4%–103.6%) and the close agreement with the gold-standard colony counting method across ten clinical samples further validate the anti-interference capability and clinical reliability of the proposed assay.

Despite these advantages, several limitations should be acknowledged. First, although the total assay time is less than 2.5 h, the procedure still involves multiple steps (e.g., magnetic separation, ligation, RCA, nicking, self-priming extension), which may require moderate operator skills. Future efforts could focus on integrating these steps into a microfluidic chip or a single-tube format to minimize manual intervention. Second, the detection limit of 2.3 CFU/mL, while highly sensitive, may be further improved by optimizing the density of the capture probe on magnetic nanoparticles or by employing digital RCA for absolute quantification. Third, the current design relies on the F23 aptamer, which is specific for *P. aeruginosa*; however, the platform is modular and can be readily adapted for other pathogens by substituting the corresponding aptamer, as noted in the introduction. Fourth, we have not yet evaluated the method for detecting viable but non-culturable (VBNC) states of *P. aeruginosa*, which may be clinically relevant in chronic infections. This represents an interesting direction for future investigation. Fifth, because the F23 aptamer recognizes surface epitopes that may persist on dead or inactivated *P. aeruginosa* cells, the current method cannot inherently distinguish between viable and non-viable bacteria. Consequently, killed bacteria could potentially generate false-positive signals. We acknowledge that dedicated control experiments using heat- or antibiotic-killed bacteria were not performed in this study; this limitation is shared by most aptamer-based detection strategies that rely on DNA amplification. When viability discrimination is clinically required, pre-treatment with a viability dye such as propidium monoazide (PMA) could be integrated to selectively crosslink DNA from dead cells and prevent their amplification, and this represents a clear direction for future optimization.

In summary, the proposed biosensing strategy successfully addresses the sensitivity, specificity, and operational simplicity requirements for perioperative detection of *P. aeruginosa*. The label-free design, isothermal amplification, and robust performance in clinical matrices position it as a promising alternative to conventional methods. With further optimization and miniaturization, this platform holds great potential for point-of-care testing in perioperative settings, as well as for broader applications in food safety and environmental surveillance.

## Conclusion

In summary, we have successfully developed a novel biosensing strategy for sensitive and label-free detection of P aeruginosa by integrating F23 aptamer-mediated target recognition, garland rolling circle amplification-triggered self-priming extension, and SG-I-based fluorescence readout. The working mechanism was systematically validated through a series of fluorescence experiments, confirming the successful assembly of each functional module and the feasibility of the cascade amplification process. Under optimized conditions, the proposed method exhibited a wide linear detection range from 10 to 10^6^ CFU/mL, with a detection limit as low as 2.3 CFU/mL, demonstrating superior sensitivity compared to conventional aptamer-based assays. The method also displayed excellent specificity against common non-target bacteria, reliable storage stability, and robust anti-interference capability in complex clinical matrices. Furthermore, validation using clinical samples yielded results highly consistent with the gold-standard colony counting method, while significantly reducing the assay time to less than 2.5 h without requiring nucleic acid extraction or thermal cycling.

Importantly, when compared with conventional PCR, our method offers distinct practical advantages. While PCR typically requires laborious nucleic acid extraction, thermal cycling equipment, and 3–4 h to complete, the proposed isothermal amplification strategy eliminates these requirements, thereby simplifying the workflow and enabling rapid point-of-care testing. Moreover, the achieved detection limit of 2.3 CFU/mL is comparable to or even better than many reported PCR-based assays for *P. aeruginosa*, yet our method maintains robust performance in complex clinical matrices such as serum without suffering from PCR inhibitors. These features collectively underscore the superior balance of sensitivity, speed, and operational simplicity offered by our platform over conventional molecular diagnostics.

The key innovations of this work lie in three aspects: (i) the label-free design circumvents the stability issues associated with covalently labeled fluorophores, offering enhanced robustness and reproducibility; (ii) the garland RCA-mediated self-priming extension strategy provides efficient signal amplification under isothermal conditions, endowing the method with a high sensitivity; and (iii) the integration of target recognition, signal amplification, and fluorescence detection into a unified platform enables straightforward operation with minimal sample pretreatment. These distinctive features render the proposed method particularly attractive for point-of-care testing in perioperative settings, where rapid, sensitive, and user-friendly diagnostic tools are urgently needed.

Looking forward, this strategy holds great promise not only for clinical diagnosis of *P. aeruginosa* infections but also for food safety inspection and environmental surveillance. Moreover, by simply substituting the corresponding aptamer, the platform can be readily adapted for the detection of other pathogenic bacteria, offering a versatile and universal approach for broad-spectrum pathogen analysis. Future efforts will focus on further simplifying the operation procedure, integrating the assay into microfluidic devices, and validating its performance in large-scale clinical cohorts to facilitate its translation from laboratory research to practical applications.

## Declarations

### Ethics Approval and Consent to Participate

This study was approved by the Medical Ethics Committee of The First People’s Hospital of Chun’an County (Approval No. 2024-04-03-12).

## Supplemental Materials

Supplementary data for this paper are available on-line only at http://jmb.or.kr.
