# Supplementary material for: Garland Rolling Circle Amplification Mediated Self-Priming Extension Strategy for Sensitive and Label-Free Pseudomonas aeruginosa Analysis in Perioperative Period
Source: J Microbiol Biotechnol. 2026 May 11;36:e2603031. doi: 10.4014/jmb.2603.03031 (PMC13173353; doi:10.4014/jmb.2603.03031)
Supplement: Supplementary file 1 [file jmb-36-e2603031-supple.pdf]

J. Microbiol. Biotechnol. 2026. 36: e2603031

https://doi.org/10.4014/jmb.2603.03031

Supplementary Table

Supplemented experimental section

Bacterial culture

Pseudomonas aeruginosa (ATCC 15442) and non-target bacterial strains including *Escherichia coli* (ATCC 25922), Staphylococcus aureus (ATCC 25923), Listeria monocytogenes (ATCC 19115), and Salmonella enterica (ATCC 14028) were obtained from Guangdong Microbial Culture Collection Center (Guangzhou, China). All bacterial strains were cultured in LB broth at 37°C with shaking at 200 rpm overnight. Bacterial cells were harvested by centrifugation at 8000 rpm for 5 min, washed twice with sterile PBS buffer (10 mM, pH 7.4), and re-suspended in the same buffer. The bacterial concentration was determined by measuring the optical density at 600 nm (OD_{600}) and further confirmed by standard plate colony counting on LB agar after incubation at 37°C for 24 h.

Table S1. Sequences of all oligonucleotides used in this study.

**Title**	**Sequences (5' to 3')**
Aptamer	CCC CCG TTG CTT TCG CTT TTC CTT TCG CTT TTG TTC GTT TCG TCC CTG CTT CCT TTC TTG-C6-
1	NH2- AC GAA CA A A AG CGA
DP	GTTCG T T AC TGC AG G G A* A*G *A* A*T *T* C*T *T* A*A *G* A*A *T* T*C *T* T* CAC TCA CA CCT GC A G TC AAC TT TTC GCT
Hp	GTG TGA GTA AA GCT AAA GT TAG TGG AAA ACC ACT AAC
